# Progress in exercise interventions for autism spectrum disorder: exploring underlying mechanisms

**DOI:** 10.3389/fpsyt.2025.1649005

**Published:** 2026-01-02

**Authors:** Chaofan Wang, Yuanxiang Zhang, Jimiao Zhu, Zhiyi Lin

**Affiliations:** 1 Minjiang University,Public Physical Education, Fuzhou, China; 2 College of Basic Medical Sciences, Fujian Medical University, Fuzhou, China; 3 College of Physical Education and Science, Fujian Normal University, Fuzhou, China; 4 School of Physical Education and Sport Science, Fujian Normal University, Fuzhou, China

**Keywords:** ASD, exercise, intervention, mechanism, review

## Abstract

ASD is a neurodevelopmental disorder with specific core symptoms. Physical activity has been demonstrated to positively influence the pathological mechanisms underlying autism and to alleviate associated symptoms. A comprehensive synthesis was conducted by reviewing and integrating relevant literature. Literature review revealed that the mechanism of physical activity intervention in autism primarily involves modulation through neuronal factors, glial cells, and gut microbiota. Neuronal factors include brain-derived neurotrophic factor, axonal protein families, and neurotransmitters. Additionally, physical activity helps alleviate stereotypical behaviors and internal anxiety in individuals with ASD, reduce obesity and cardiovascular diseases in some patients, and enhance social communication skills.

## 1 Introduction

Autism, also referred to as autism spectrum disorder (ASD), is a neurodevelopmental disorder with specific core symptoms. It is marked by distinct cognitive traits that influence information processing and behavior, and is frequently accompanied by various comorbidities (1). Due to a combination of environmental factors and strong genetic predisposition, most children with ASD exhibit intellectual disabilities, while a minority may display elevated aggression, sensory impairments, emotional dysregulation, stereotyped behaviors, and deficits in social communication (2). The core characteristics of ASD are primarily associated with neurological and cognitive dysfunctions. Among the common comorbid conditions are attention-deficit/hyperactivity disorder (ADHD), attentiondeficit disorder (ADD), anxiety, depression, and epilepsy (3). Individuals with ASD often experience reduced motor proficiency, primarily due to prolonged sedentary behavior and insufficient physical activity. In some cases, muscular dystrophy may also be present (4).

According to newly released 2025 data from the U.S. Centers for Disease Control and Prevention (CDC). ASD is diagnosed in 1 out of every 31 children in the United States, corresponding to a prevalence of 3.2%. This marks an increase from 2.7% in 2020 and reflects a continually escalating public health concern (5). Current treatment strategies for ASD primarily include pharmacological interventions, such as psychotropic medications, and non-pharmacological approaches, such as behavioral therapies. However, pharmacological treatments are frequently associated with long-term adverse effects, whereas non-pharmacological therapies can be both costly and time-consuming. Against this backdrop, exercise interventions have emerged as a feasible adjunctive approach for ASD, offering advantages such as low cost, flexibility, and ease of implementation compared with conventional therapies (6). Wen et al. reported that sensory-integration exercise training reduced the average Social Responsiveness Scale (SRS-2) score in children with ASD from 98.4 to 85.2—a net decrease of 13.2 points—indicating improvements in social awareness and communication, as well as reduced social avoidance (7). Tse et al. further demonstrated that cycling training significantly increased Tower of London scores (from 11.00 to 18.59) and markedly reduced Stroop interference scores, suggesting enhanced planning ability, executive functioning, and cognitive flexibility (8).Collectively, these studies indicate that exercise can effectively mitigate ASD symptom severity and improve motor performance, stereotyped behaviors (self-stimulating behaviors, SSB), social communication, executive function, mental health, and a range of cognitive outcomes in individuals with ASD (9–11).

However, current research primarily focuses on evaluating the efficacy of exercise interventions for ASD; however, the underlying neurochemical mechanisms and the distinct impacts of various forms of exercise have not been thoroughly investigated. In addition, recent advances in gut microbiota research have revealed increasingly robust associations between gut flora and neuropsychiatric disorders, with growing evidence suggesting its potential significance in ASD (12). This study seeks to provide an in-depth analysis of the specific mechanisms and feasibility of exercise-based interventions for ASD, with a particular focus on neurochemical pathways and gut microbiota, grounded in a comprehensive review of recent literature.

## 2 Methods

### 2.1 Search strategy

A comprehensive search was performed in the Web of Science database, PubMed, in November 2025, using keywords aligned with the study objectives: “autism,” “ASD,” “sport,” “exercise,” “physical activity,” “exercise intervention,” and “mechanism.” The following search strategy was constructed: ((autism OR ASD) AND (sport OR exercise OR physical)) AND (mechanism). A total of 2006 articles were retrieved.

### 2.2 Inclusion and exclusion criteria

The inclusion criteria were as follows (1): the study population consisted of individuals diagnosed with ASD (2); the study involved an exercise-based intervention; (3) the research focused on physiological mechanisms related to exercise and ASD; (4) the intervention procedures and study outcomes were clearly described; (5) the article was indexed in academic databases.

The exclusion criteria included: (1) non–peer-reviewed publications, such as editorials, conference abstracts, or dissertations; (2) articles not published in English.

**Figure 1:**
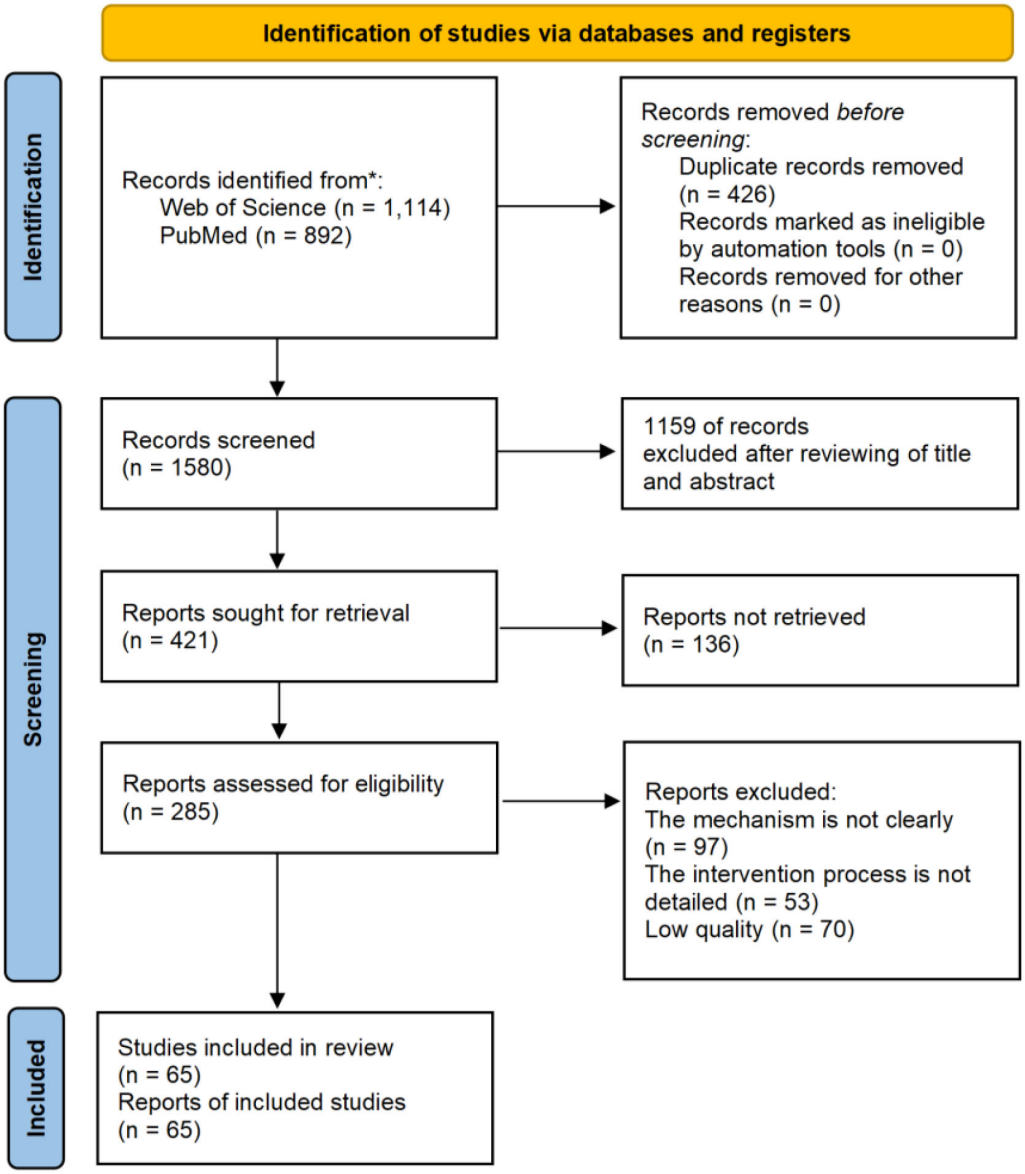
Flow chart indicating the stages of article selection in this review (PRISMA 2020).

After conducting the search, the authors independently screened the retrieved studies to assess whether they met the eligibility criteria. Studies lacking sufficient information were subjected to full-text review to confirm their inclusion. Two authors independently evaluated the studies based on the predetermined inclusion and exclusion criteria. Subsequently, the authors jointly reviewed the selected studies until a consensus was reached regarding their inclusion.After removing duplicate records, a total of 1,580 articles remained. Screening of titles and abstracts resulted in the exclusion of 1,159 articles. Full-text assessments were then conducted to determine eligibility, and the reference lists of the included studies were additionally examined to identify further relevant publications. Ultimately, 65 studies met the inclusion criteria. A flow diagram summarizing the study selection process is provided in **Figure 1**.

## 3 Mechanisms of exercise based interventions in ASD

Exercise has been shown to modulate the pathophysiology of ASD by influencing neuronal factors, glial cell activity, and the composition of the gut microbiota (13, 14). Key molecular pathways implicated in neuronal and cortical organization include growth factors, neurotrophic factors such as brain-derived neurotrophic factor (BDNF), and neurotransmitters. Activity dependent structural remodeling enables neuronal migration and morphological changes, leading to synapse formation and integration into functional neural networks necessary for normal brain function (13). Impairments in synapse formation and synaptic plasticity contribute to functional and cognitive deficits, constituting a core pathological mechanism in ASD. Neuron-related factors are thought to alleviate symptoms by enhancing synaptic plasticity (15). The beneficial mechanisms of exercise interventions in ASD primarily involve neurotrophic factors, synaptic proteins, serotonin, and glial cell activity.

Clinical intervention studies have shown that short-duration, low-to-moderate-intensity exercise (50–65% of maximum heart rate) can reduce stereotyped behaviors in children and adolescents with ASD for up to one hour. Moderate-intensity exercise has been reported to alleviate anxiety and tension in individuals with ASD, whereas high-intensity exercise yields little benefit and may even exacerbate anxiety. Regular moderate-intensity aerobic exercise can mitigate age-related loss in brain volume and increase the volume of regions responsible for cognition, attention, and memory, an effect that appears to be mediated through regulation of BDNF (16, 17). Moreover, 12–24 weeks of aerobic or combined (aerobic + resistance) exercise performed 3–5 times per week for 20–40 minutes at an intensity of 40–60% VO_2_ max has been shown to reduce the frequency of repetitive behaviors (e.g., hand flapping, spinning) by 40–60%, decrease distractibility, and enhance sustained attention during tasks such as puzzles or reading. Following the intervention, participants also exhibited increased abundances of beneficial gut bacteria, including Bifidobacterium and Faecalibacterium prausnitzii, accompanied by reductions in harmful and pathogenic taxa (18).

In animal studies, King et al. reported that 4 weeks of moderate-intensity aerobic exercise performed five times per week increased BDNF expression in the dorsal hippocampus and enhanced antioxidant capacity, thereby improving cognitive flexibility and motor function in ASD model rats (19). Similarly, Peng et al. demonstrated that eight weeks of moderate-intensity aerobic exercise, conducted 3–5 times per week for 40 minutes per session, modulated signaling along the microbiota–gut–brain axis, enhanced central nervous system function, and upregulated 5-HT and BDNF expression, leading to improvements in cognitive function and stereotyped behaviors in ASD models (20). Together, these clinical and animal studies suggest that low-to-moderate intensity, frequent exercise confers beneficial effects on ASD-related symptoms. However, while animal studies of exercise interventions in ASD have become increasingly systematic, clinical research remains limited in number and detail, highlighting the urgent need for targeted human studies.

### 3.1 Exercise induced modulation of brain-derived neurotrophic factor in ASD

BDNF is a critical neurotrophin primarily expressed in the hippocampus. Once synthesized and secreted, BDNF regulates enzymatic activity via dendritic mechanisms. These processes, in turn, influence synaptic development, plasticity, and neuronal growth. An et al. examined the role of the long 3′ untranslated region (UTR) in the dendritic targeting of BDNF mRNA using a mouse mutant selectively expressing the long 3′ UTR of BDNF mRNA (21). Mice deficient in this long 3′ UTR variant exhibited impaired dendritic localization, resulting in diminished synaptic plasticity in hippocampal neurons. These findings suggest that dendritic synthesis and secretion of BDNF are essential for synaptic plasticity, and that deficits in BDNF signaling impair synaptic development and neuronal growth, suggesting a potential molecular mechanism underlying the pathogenesis of ASD.

**Figure 2:**
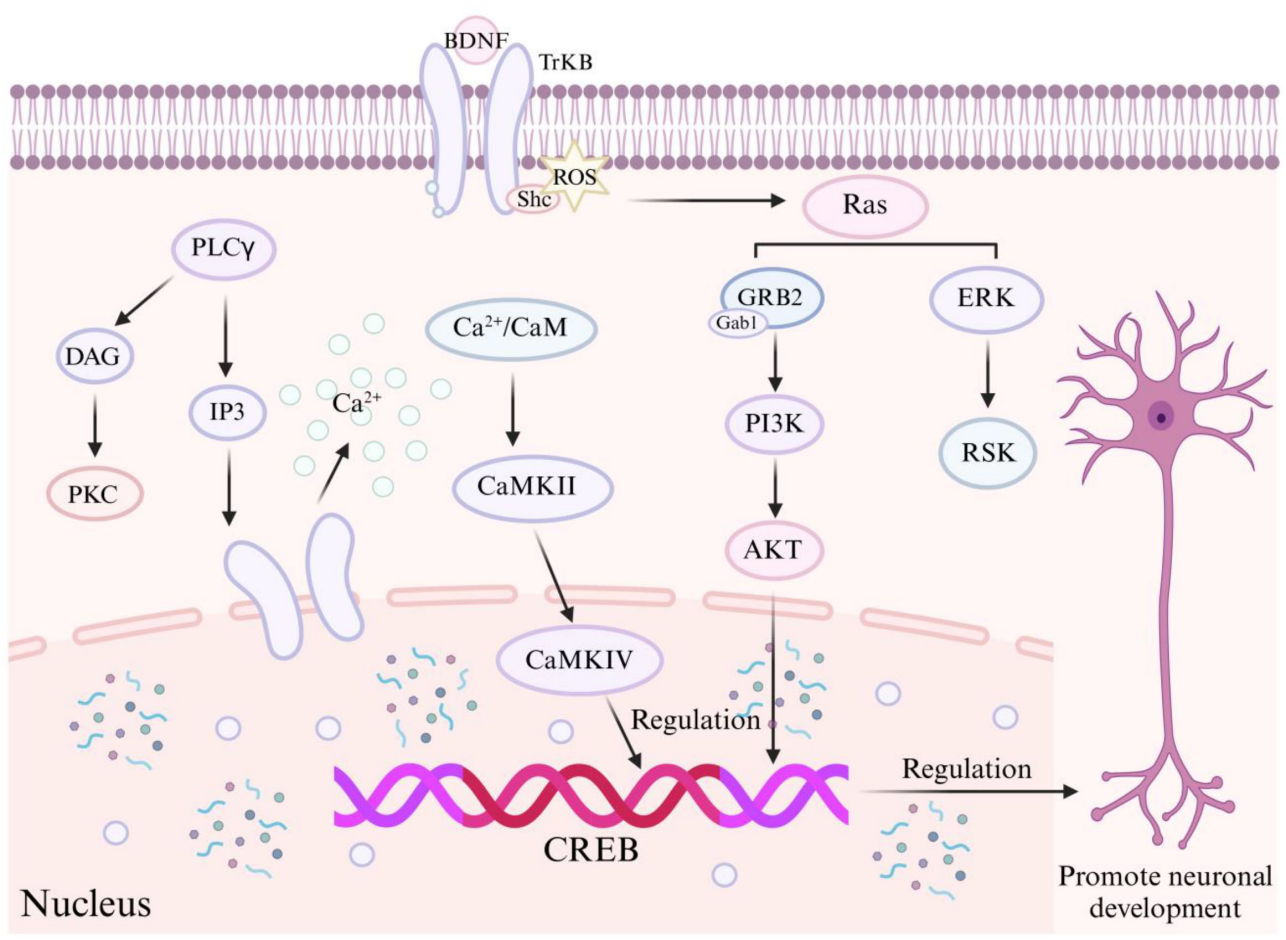
Mechanism of BDNF in ASD. BDNF, brain-derived neurotrophic factor; PLC, phospholipase C; TrkB, tyrosine kinase receptor B; GRB2, growth factor receptor bound protein 2; PI3K, the phosphoinositide 3-kinase; AKT, protein kinase B; ERK, extracellular regulated protein kinases; RSK, ribosomal S6 kinase; IP3, trisphosphate; DAG, diacylglycerol; CaMKII, Ca^2+^ /calmodulin dependent protein kinase II; CaMKIV, Ca^2+^ /calmodulin dependent protein kinase IV; PKC, protein kinase C; CREB, cAMP response element-binding protein.

Exercise has been shown to improve cognitive flexibility and motor function and to alleviate ASD-related symptoms. These effects are mediated by regulating BDNF expression, which activates the BDNF/TrkB signaling pathway and its downstream cascades and modifies astrocyte morphology (19–23). Studies have shown that four weeks of moderate-intensity aerobic exercise—five sessions per week, 30 minutes per session (10 min at 8.3 cm/s, 10 min at 13.3 cm/s, and 10 min at 26.6 cm/s)—enhances hippocampal BDNF and IL-6 levels, as well as skeletal muscle antioxidant enzymes, thereby improving cognitive flexibility and motor function in ASD model rats (19). Furthermore, emerging evidence indicates that 30 days of voluntary aerobic exercise, performed without forced intervention to avoid stress-induced effects on BDNF levels, can similarly confer beneficial effects. Induced elevation of BDNF levels may be mediated by the release of endogenous histone deacetylase (HDAC) inhibitors triggered by exercise. Aerobic exercise alters hepatic metabolism, facilitating the transport of the ketone body D-β-hydroxybutyrate (DBHB) through the bloodstream to the brain, where it functions as a class I HDAC inhibitor in the hippocampus, particularly targeting HDAC2 and HDAC3. Treatment of primary neurons with DBHB reduces the binding of HDAC2 and HDAC3 to the BDNF promoter, thereby increasing BDNF expression (22). Additionally, exercise mitigates ASD related symptoms by modulating the BDNF/TrkB signaling pathway (23). Following its expression and secretion, BDNF binds to its high-affinity receptor, TrkB, a tyrosine kinase receptor containing multiple tyrosine residues within its intracellular domain. Phosphorylation of TrkB subsequently initiates several key intracellular signaling cascades, including the mitogen activated protein kinase/extracellular signal-regulated kinase (MAPK/ERK) pathway, the phosphoinositide 3-kinase (PI3K) pathway, and the phospholipase C (PLC) pathway (24). (**Figure 2**).

#### 3.1.1 MAPK/ERK signaling pathway

Phosphorylated TrkB acts as the initiating trigger for the activation of the MAPK/ERK signaling pathway. Within this cascade, MAPK/ERK kinases phosphorylate and activate the transcription factor CREB (cAMP response element-binding protein). Upon phosphorylation, CREB translocates into the nucleus, where it regulates gene transcription by binding to promoter regions. This transcriptional activity promotes cellular survival, differentiation, and proliferation.

#### 3.1.2 PI3K signaling pathway

Upon binding to the Tyr515 residue, the Ras complex initiates the activation of multiple signaling cascades, including the PI3K/Akt and MEK/MAPK pathways. Activation of the PI3K/Akt pathway regulates the expression and function of proteins critical for neuronal survival, growth, and differentiation.

#### 3.1.3 PLCγ signaling pathway

PLCγ Signaling Pathway:Phosphorylation of the Tyr816 residue on the TrkB receptor activates the PLCγ signaling pathway, resulting in the production of inositol trisphosphate (IP3) and diacylglycerol (DAG). Activation of the IP3 pathway by PLCγ induces calcium release from the endoplasmic reticulum, which subsequently activates CaMKII (Ca²^+^ /calmodulin dependent protein kinase II), leading to the phosphorylation of the transcription factor CREB. Meanwhile, DAG activates protein kinase C (PKC). Activation of both CaMKII and PKC plays a crucial role in promoting cell survival, neurite outgrowth, and synaptic plasticity.

These signaling pathways are critical for neurogenesis, the functional and structural integrity of neurons, and synaptic plasticity, thereby influencing the pathogenesis of ASD (25).

### 3.2 Exercise induced the modulation of neurexin family in ASD

The neurexin (NRXN) family, encoded by the homologous genes NRXN1, NRXN2, and NRXN3, constitutes a group of presynaptic transmembrane proteins. Each gene gives rise to two principal isoforms: the longer α-neurexins and the shorter β-neurexins, which are transcribed from alternative promoters. Further diversity within the NRXN repertoire is generated through alternative splicing (AS) of both isoforms, resulting in the production of more than a thousand distinct splice variants. Owing to the presence of multiple alternatively spliced exons across all three NRXN genes, a vast array of isoform diversity is achieved. All α-NRXNs undergo alternative splicing at six canonical splice sites (SS1–SS6), while β-NRXNs are limited to splicing at SS4 and SS5. The resulting splice variants exhibit selective affinities for a broad range of postsynaptic ligands, contributing to the regulation of synaptic specification, plasticity, and strength. Notably, inclusion of exon SS4 leads to the SS4^+^ variant, incorporating a 30-amino-acid insertion, whereas exon skipping yields the SS4^−^ variant. The presence or absence of this exon critically determines the binding specificity of neurexins to distinct postsynaptic molecules, thereby enhancing synaptic differentiation, plasticity, and specificity, as well as behavioral traits. These mechanisms are believed to underlie the potential amelioration of cognitive deficits observed in individuals with ASD (26).

Exercise can promote NRXN1–3 expression at alternative splice site 4 (SS4), thereby increasing the abundance of SS4^+^ splice variants in the prefrontal cortex and enhancing synaptic differentiation, plasticity, and specificity, ultimately contributing to the alleviation of ASD-related symptoms (25, 26). In support of this mechanism, a 12-week progressive aerobic exercise program—performed three times per week with treadmill speed gradually increased from 10 to 32 revolutions per minute (RPM)—has been shown to upregulate both the α- and β-isoforms of NRXN1–3 at SS4, thereby increasing the abundance of SS4^+^ splice variants in the prefrontal cortex (26). Notably, this effect was not observed in the hippocampus, likely due to greater molecular sensitivity and structural plasticity in the prefrontal cortex compared to the hippocampal region. Using splice site–specific primers, researchers detected significant exercise induced alternative splicing changes at SS4 of NRXN1–3 exclusively in the prefrontal cortex following continuous progressive exercise. These findings were further corroborated by quantitative PCR (qPCR), which confirmed an upregulation of SS4^+^ splice variants across all NRXN genes in the frontal cortex of continuous progressive trained mice (27).

### 3.3 Exercise induced modulation of neurotransmitters in ASD

Autistic individuals exhibit numerous abnormalities in their neurotransmitter systems, including serotonin, GABA, glutamate, dopamine, opioids, and oxytocin, with serotonin being the most significant. Serotonin, also known as 5-hydroxytryptamine (5-HT), plays a crucial role in brain development and alleviating ASD through the regulation of trophic factors (28). For example, 5-HT1A mediates the release of BDNF, which subsequently alleviates ASD. Serotonin is primarily produced by enterochromaffin cells and plays a pivotal role in secretion, sensation, and signal transduction within the body. It is a key signaling molecule in the “microbiota-gut-brain axis” and can also influence CNS microglial cells, thereby affecting mood and behavior in individuals (29).

The role of 5-HT, commonly known as serotonin, in ASD has been extensively documented. Empirical evidence suggests that repetitive behaviors, stereotyped movements, and deficits in social interaction—core features of ASD—are regulated by 5-HT signaling (30). This signaling involves the release of serotonin from neuronal vesicles into the synaptic cleft and extracellular space, where it binds to 5-HT receptors, including both heteroreceptors and autoreceptors (31). Following the release of 5-HT, the primary regulatory mechanism controlling extracellular 5-HT levels is the reuptake mediated by transporters, among which the 5-HT transporter (SERT) exhibits the highest affinity for 5-HT. During early development, 5-HT, through its interactions with enzymes, receptors, and transporters, coordinates essential neuronal processes, including cell division, differentiation, migration, and synaptogenesis, playing a pivotal role in the proper patterning and organization of sensory cortical neurons. Evidence from both human and rodent studies suggests that alterations in the synthesis, release, signaling, uptake, or metabolism of 5-HT can lead to dysregulated serotonergic activity, which may contribute to the risk or severity of ASD symptoms (32). Additionally, as one of the precursors of 5-HT, the level of tryptophan (TRP) has been shown to directly influence serotonin levels in the central nervous system (CNS) (33).

Exercise has been shown to increase levels of 5-HT in the CNS across species. This elevation is influenced by several factors, including increased levels of L-tryptophan, enhanced presynaptic 5-HT release, and reduced activity of monoamine oxidase (MAO), the enzyme responsible for the breakdown of monoamines in the synaptic cleft (34). A 4-week aerobic exercise regimen—performed once daily for 30 minutes with progressively increasing intensity (5 to 15 m/min, treadmill incline 0°)—can further enhance serotonin synthesis by activating tryptophan hydroxylase (TPH), a rate-limiting enzyme in serotonin biosynthesis, and by stimulating serotonin 1A (5-HT_1_A) receptors, thereby ameliorating ASD-related symptoms (35). Studies have shown that two weeks of moderate-intensity aerobic exercise, performed for 20 minutes per day, exerts protective effects on vascular and neural function in rats, whereas high-intensity exercise downregulates neurotrophins and disrupts the expression of cell cycle–related proteins. In contrast, low-to-moderate–intensity aerobic exercise elevates 5-HT levels, modulates GABA and glutamate concentrations to maintain excitatory–inhibitory balance, and reduces the expression of inflammatory cytokines such as TNF-α, IL-1β, and IL-6, collectively contributing to the improvement of ASD-related symptoms (36). Moreover, aerobic exercise increases plasma concentrations of free fatty acids, which bind to albumin and displace TRP from its binding sites. Since the uptake of TRP by the brain and other organs primarily depends on the proportion of free (unbound) TRP, it is hypothesized that a higher fraction of free TRP crosses the blood–brain barrier (BBB), thereby enhancing cerebral serotonin synthesis (37, 38).

### 3.4 Exercise induced regulation of glial cell function in ASD

Growing evidence suggests that glial cells in the brains of individuals with ASD play pivotal roles in the progression and prognosis of the disorder. These cells interact dynamically with neurons via both chemical signals (e.g., neurotransmitters, neurotrophic factors, and cytokines) and physical mechanisms (e.g., gap junctions), thereby maintaining and modulating neuronal function. Consequently, glial cells function not only as key modulators of neuronal activity during brain development, but also as critical regulators of disease pathophysiology (39).

Astrocytes, the most abundant glial cells in the brain, are essential for supporting neuronal function during development. They regulate synaptic neurotransmitter levels by modulating dendritic spine formation and neuronal migration, thereby facilitating synaptogenesis and maintaining synaptic integrity. In ASD, astrocytic dysfunction may disrupt neurotransmitter metabolism and impair synapse formation. Consequently, alterations in astrocyte number or function may underlie the connectivity deficits commonly observed in individuals with ASD (40). Microglia, the brain’s resident immune cells, contribute to neurodevelopment, synaptic plasticity, and cognitive function. In ASD, elevated mitochondrial DNA (mtDNA) in serum extracellular vesicles has been shown to activate human microglia, leading to the release of the proinflammatory cytokine interleukin-1β (IL-1β) (41). Neuroimaging studies in individuals with ASD have demonstrated widespread microglial activation across multiple brain regions. Given that IL-1β impairs synaptic plasticity and neurogenesis, such immune activation may contribute to the emergence of stereotyped behaviors and cognitive deficits in ASD.

**Figure 3:**
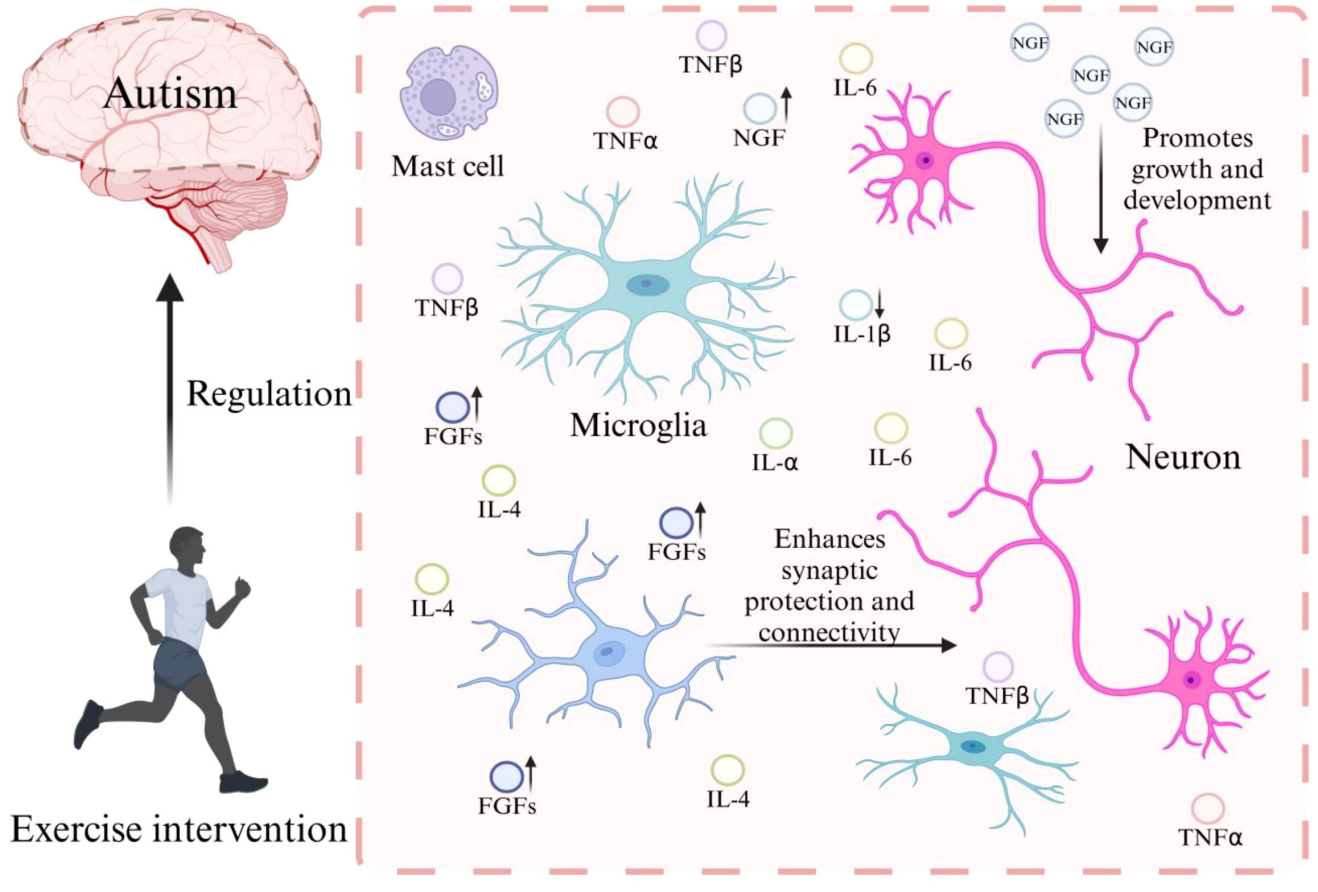
Mechanism of exercise-induced glial cell modulation in ASD. FGFs, fibroblast growth factors; NGF, nerve growth factor; TNF-α, tumor necrosis factor α; TNF-β, tumor necrosis factor β; IL-1β, interleukin-1 β; IL-α, interleukin-1 α; IL-4, interleukin-4; IL-6, interleukin-6.

Exercise can elevate the expression of fibroblast growth factors (FGFs) and nerve growth factor (NGF), activate the mTOR signaling pathway in astrocytes, regulate microglial activation, and increase astrocyte density, thereby improving brain functions such as learning and memory and normalizing microglial metabolism and function, ultimately alleviating ASD-related symptoms (42–48). Studies show that a 4-week light-to-moderate aerobic exercise program—performed five times per week, 30 minutes per session, with a speed of 4–6 m/min—can increase astrocyte density in mice (42). This elevation has been linked to higher levels of exercise-induced FGFs and NGF (43, 44), both of which are known to promote astrocyte proliferation (45, 46). Lloyd et al. conducted a 6-week aerobic exercise program, performed five times per week for 12 hours per session, with running speed not exceeding 17 m/min, an average running bout duration of 2.04 ± 1.95 minutes, and inter-bout rest periods ranging from 0.33 to 30 minutes. They demonstrated that aerobic exercise enhances mTOR signaling in astrocytes, which is critical for brain functions such as learning, memory, cell growth, proliferation, and survival (47) (**Figure 3**). Moreover, a 10-day aerobic exercise program, starting at 25 minutes and increasing by 5 minutes every two days, performed at moderate intensity (first 5 minutes at 6.2 m/min, next 10 minutes at 8.2 m/min, followed by 10–15 minutes at 9.2 m/min, then 5–10 minutes at 10.2 m/min, and the final 5 minutes at 11.2 m/min, treadmill incline 0°), can modulate microglial activation (48). This effect reduces age related increases in hippocampal interleukin-1β (IL-1β) and helps alleviate ASD related symptoms (49). In this study, older sedentary mice expressed elevated levels of microglial markers, whereas exercise significantly decreased their expression. These findings suggest that exercise associated cognitive improvements may result from the normalization of microglial metabolism and function.

### 3.5 Exercise induced modulation of gut microbiota in ASD

ASD is a neurodevelopmental condition of the CNS that typically emerges in early childhood. Growing evidence suggests that alterations in the gut microbiota are associated with increased symptom severity. The gut microbiota plays a pivotal role in modulating the gut–brain axis, potentially influencing neurodevelopmental processes and behavioral outcomes relevant to ASD (50). Recent studies also indicate that gut microbial composition can affect both cognitive and behavioral functions (51). Disruptions in the microbiota–gut–brain axis, especially microbial imbalances, are linked to neurobehavioral abnormalities and gastrointestinal dysfunction in ASD (52, 53).

The gut–brain axis refers to the bidirectional communication network between the gut and the brain, primarily mediated by the vagus nerve (54). Through this pathway, the vagus nerve interacts with the gut microbiota within the enteric nervous system (ENS), transmitting critical signals to the CNS, which processes the input and initiates appropriate responses (55). Studies have shown that the gut microbiota influences the gut–brain axis via neuroendocrine and metabolic pathways. It also directly interacts with the ENS, supporting the structural and functional development of the nervous system (56). These mechanisms affect brain function and may contribute to the development and progression of ASD (57).

**Figure 4:**
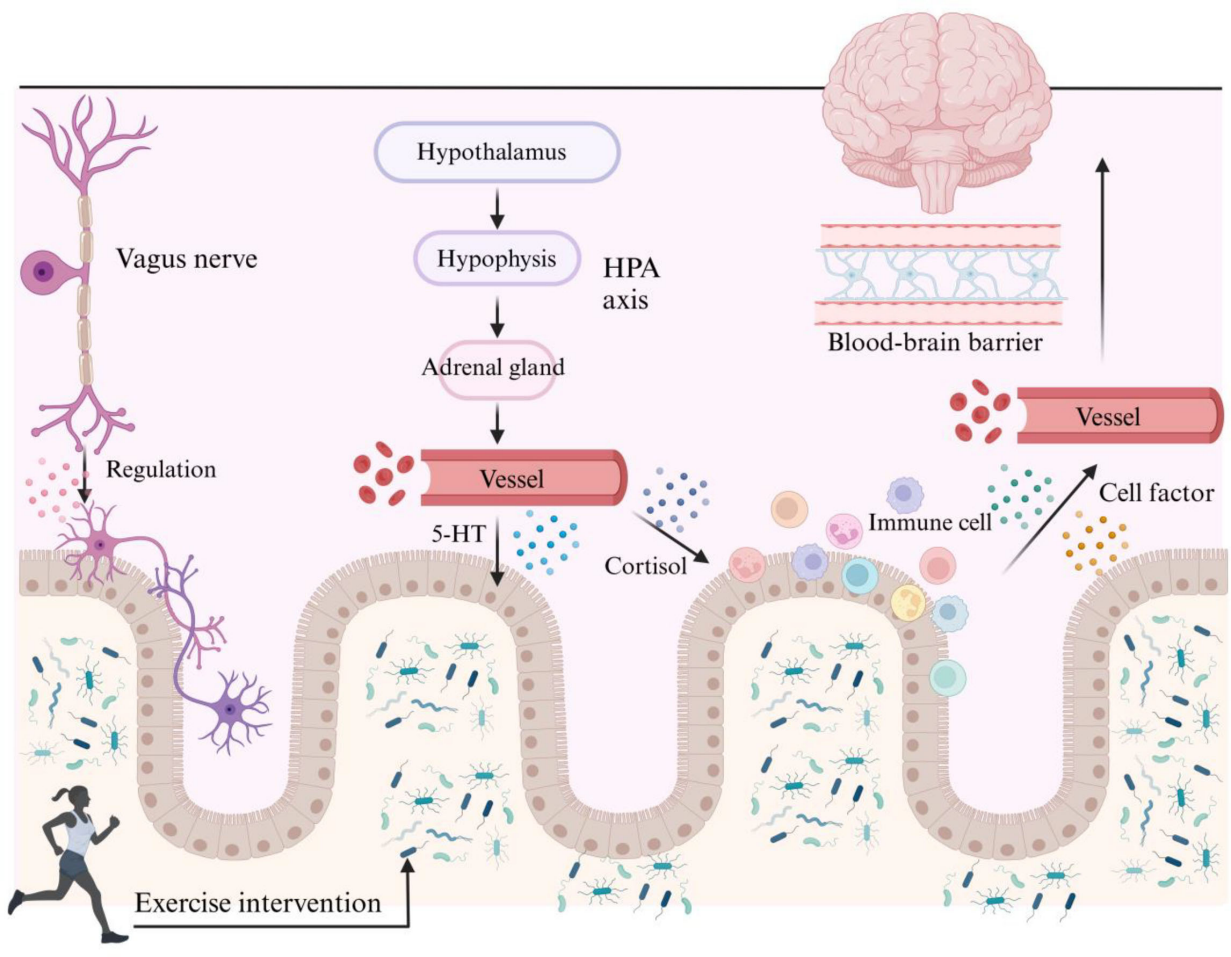
Mechanism of exercise-induced gut microbiota modulation in ASD. 5-HT, 5-hydroxytryptamine; HPA, hypothalamic-pituitary-adrenal.

Exercise can increase gut microbiota diversity, enrich beneficial bacteria such as butyrate-producing species, and modulate signaling along the microbiota–gut–brain axis. Concurrently, it elevates the expression of 5-HT, BDNF, and GABA, thereby regulating neurotransmitter balance, improving central nervous system function, enhancing neuronal antioxidant capacity and functionality, and ultimately contributing to the amelioration of cognitive performance, motor abilities, and stereotyped behaviors in individuals with ASD (18, 20). Recent studies have shown that an 8-week program of moderate-intensity aerobic exercise, performed 3–5 times per week for 40 minutes per session, can increase gut microbiota diversity, enrich beneficial bacteria, and reduce the expression of pro-inflammatory cytokines such as TNF-α, IL-1β, and IL-6. This intervention also attenuates excessive microglial activation and modulates signaling along the microbiota–gut–brain axis, thereby improving central nervous system function. Additionally, exercise enhances the activity of antioxidant enzymes such as SOD and GSH-Px, decreases ROS levels, elevates 5-HT and BDNF expression, and strengthens neuronal antioxidant capacity and functionality (20). Moreover, an 8-week program of combined exercise (aerobic plus resistance) or aerobic exercise, performed at least twice per week for 30–60 minutes per session at moderate intensity (60–75% Heart Rate/VO_2_max), can optimize gut microbiota composition, enrich beneficial butyrate-producing bacteria such as *Faecalibacterium prausnitzii* and *Roseburia hominis*, and increase microbial diversity. This intervention also elevates GABA levels and regulates neurotransmitter balance, thereby exerting positive effects on cognitive function, motor performance, and stereotyped behaviors in individuals with ASD (18). (**Figure 4**) Studies of the gut microbiome have found that athletes exhibit greater microbial diversity compared to nonathletes. This effect is likely attributed to the enhanced metabolic flexibility induced by regular physical activity. Exercise-derived metabolites can act as energy sources or nutrients for specific microbial taxa. They may also exert physiological effects that shape microbial composition and promote microbial diversity in the gut. The study indicates that moderate-intensity aerobic exercise (60–75% Heart Rate/VO_2_max), performed three times per week for 8–12 weeks, is relatively effective (58, 59).

## 4 Discussion

A comprehensive review of the literature on exercise interventions for ASD indicates that physical activity can effectively alleviate ASD related symptoms. The beneficial effects of exercise are primarily mediated through the modulation of neuronal function, glial cell activity, and gut microbiota composition. Neuron-related mechanisms enhance synaptic plasticity, while alterations in the gut microbiota influence symptoms via the microbiota–gut–brain axis by promoting improved gut–brain communication through vagal pathways. Importantly, different forms of exercise yield distinct therapeutic effects, and individuals with ASD may benefit from selecting exercise modalities tailored to their specific needs and physiological profiles.

Although this review does not primarily focus on exercise intervention programs, it summarizes relevant exercise-based approaches for ASD. Multiple studies have confirmed that different types of low-to-moderate-intensity exercise interventions can exert positive effects on nervous system function and individuals with ASD by modulating neural pathways, neurotransmitters, and gut microbiota. Fahimi A et al. found that a 5-week intermittent aerobic exercise combined with voluntary aerobic activity (treadmill training 5 days per week plus running wheel access 7 days per week, moderate intensity, starting at 8 m/min and increasing by 2 m/min each week) significantly increased BDNF expression, altered astrocyte morphology, elevated TrkB receptor levels, and activated the BDNF/TrkB signaling pathway (23). Moreover, Mahalakshmi B et al. demonstrated that a 2-week program of moderate-intensity aerobic exercise, performed 20 minutes per day in rats, protects vascular and neural function, increases 5-HT levels, regulates the balance between GABA and glutamate, and maintains excitatory–inhibitory homeostasis in the brain (36). Saur L et al. found that a 4-week program of light-to-moderate aerobic exercise, performed five times per week for 30 minutes per session at a speed of 4–6 m/min, upregulates the expression of FGFs and NGF and increases astrocyte density in mice (42). In studies conducted on individuals with ASD, Nakhal MM et al. further reported that an 8-week program of combined exercise (aerobic plus resistance) or aerobic exercise alone, performed at least twice per week for 30–60 minutes per session at moderate intensity (60–75% Heart Rate/VO_2_max), not only optimizes gut microbiota composition—by enriching beneficial butyrate-producing bacteria and increasing microbial diversity—but also elevates GABA levels and regulates neurotransmitter balance, thereby exerting positive effects on cognitive function, motor performance, and stereotyped behaviors in individuals with ASD (18). These interventions generally fall into four categories: aerobic exercise, resistance training, whole body vibration training, and combined modalities. For instance, performing aerobic exercise such as jogging for 15–20 minutes before class has been shown to enhance learning abilities in children with ASD (60). Resistance training may alleviate executive function deficits by improving muscular strength (61). Whole body vibration training has been reported to increase bone density and physical activity levels in children (62). Additionally, combining exercise with other therapeutic modalities can further mitigate ASD symptoms. For example, integrating music and physical activity may positively influence both physiological and psychological outcomes (63). Based on these programs, several key principles have emerged (1): exercise should be performed multiple times daily (16) (2); each session should be brief (64); (3) intensity should be kept at a low-to-moderate level (17); (4) combining exercise with other therapies may enhance overall treatment efficacy. The specific exercise protocols are as follows: for children and adolescents with ASD, a 12-week program is recommended, consisting of moderate-intensity aerobic exercise (such as brisk walking, swimming, cycling, running, or yoga) performed three times daily for 10–15 minutes, five days per week, combined with resistance training twice per week (using elastic bands or bodyweight exercises). For ASD patients with comorbid obesity, it is suggested to extend the duration of aerobic exercise to 20–30 minutes per session. For individuals who are untrained or elderly, an effective regimen is an 11-week program of regular vibration training using a low-intensity whole-body vibration device, performed 2–5 times per week for 5–8 minutes per session (65).

Exercise not only alleviates stereotyped behaviors and internal anxiety in individuals with ASD but also mitigates comorbid conditions such as obesity and cardiovascular disease, while enhancing social communication skills. However, current research has several limitations: (1) although animal studies on underlying mechanisms are relatively systematic, clinical studies remain limited in number and detail, and lack stratified analyses based on age (e.g., older ASD patients), symptom severity, or comorbidities; (2) existing studies are still fragmented and lack systematic investigation, highlighting the need to clarify the mechanisms underlying exercise interventions, with particular attention to the role of gut microbiota and its influence on intervention outcomes; (3) while exercise principles have been proposed, individualized protocols for different comorbidities remain insufficiently detailed and require further exploration; and (4) although studies have examined neurons, glial cells, and gut microbiota, the interactions among these factors remain to be elucidated in future research.
